# The Florey Adelaide Male Ageing Study (FAMAS): Design, procedures & participants

**DOI:** 10.1186/1471-2458-7-126

**Published:** 2007-06-27

**Authors:** Sean A Martin, Matthew T Haren, Sue M Middleton, Gary A Wittert, Members of the Florey Adelaide Male Ageing Study (FAMAS)

**Affiliations:** 1 Discipline of Medicine, University of Adelaide, Adelaide, SA, 5000, Australia; 2 Spencer Gulf Rural Health School, University of South Australia, Whyalla-Norrie, SA, 5608, Australia; 3 Discipline of Public Health, University of Adelaide, Adelaide, SA, 5000, Australia

## Abstract

**Background:**

The Florey Adelaide Male Ageing Study (FAMAS) examines the reproductive, physical and psychological health, and health service utilisation of the ageing male in Australia. We describe the rationale for the study, the methods used participant response rates, representativeness and attrition to date.

**Methods:**

FAMAS is a longitudinal study involving approximately 1200 randomly selected men, aged 35–80 years and living in the north – west regions of Adelaide. Respondents were excluded at screening if they were considered incapable of participating because of immobility, language, or an inability to undertake the study procedures. Following a telephone call to randomly selected households, eligible participants were invited to attend a baseline clinic measuring a variety of biomedical and socio-demographic factors. Beginning in 2002, these clinics are scheduled to reoccur every five years. Follow-up questionnaires are completed annually. Participants are also invited to participate in sub-studies with selected collaborators.

**Results:**

Of those eligible to participate, 45.1% ultimately attended a clinic. Non-responders were more likely to live alone, be current smokers, have a higheevalence of self-reported diabetes and stroke, and lower levels of hypercholesterolemia. Comparisons with the Census 2001 data showed that participants matched the population for most key demographics, although younger groups and never married men were under-represented and elderly participants were over-represented. To date, there has been an annual loss to follow-up of just over 1%.

**Conclusion:**

FAMAS allows a detailed investigation into the effects of bio-psychosocial and behavioural factors on the health and ageing of a largely representative group of Australian men.

## Background

The well documented needs of an ageing population have lead to an increased demand for research examining the determinants and conditions that promote healthy ageing [1,2]. Longitudinal studies have been identified as an important source of information on the ageing process through their unique ability to track a variety of events and conditions and the interactions between them over an extended period of time [3]. A large number of cohort studies now exist, both in Australia and abroad (see [4] for review), that to a varying extent have sought to address issues associated with the ageing process.

Despite this, there still remain few comprehensive studies investigating the biomedical physical, psychological, social and behavioural elements of the ageing process in Australian men, in spite of poorer health outcomes when compared to ageing women [5]. Compared to women, Australian men have higher rates of physical and psychological disease, and death. Generally, men are also less likely than women to adopt a healthier lifestyle [6]. Despite these disparities, the health of men and changes in their health status with ageing is one of the most understudied areas of health research. This has lead to a number of peak and government bodies to call for a comprehensive men's health longitudinal study [7-9]. Accordingly, a well-characterised cohort of men representing a range of age groups, and followed for an extended period, is considered of value.

The Florey Adelaide Male Ageing Study (FAMAS) is a multi-disciplinary population cohort study examining the health and health-related behaviours of men, aged 35–80 years and living in the north-west regions of Adelaide. It employs a broad range of investigative procedures in assessing the variable interactions that contribute to the health and health related behaviours of men. The principal aims of FAMAS are to investigate: 1) Incidence of and risk factors associated with chronic physical and psychological disorders in a representative group of Australian men; 2) Endocrinology of the ageing male and its relationship with age, health status and male-specific conditions (e.g. prostate health, erectile function, lower urinary tract symptoms); 3) Determinants of the utilization of health services amongst males. The main outcome variables are incidence of chronic health conditions (e.g. diabetes, cardiovascular disease, asthma, musculoskeletal conditions, cancer, obesity); sexual health and function; prostate health and function (including lower urinary tract symptoms); muscle strength and function; psychological disorders and function, male health service utilization, general health and well-being; physical activity levels; medication usage. In addition, the project involves a number of cross-sectional sub-studies investigating specific age related conditions.

This paper details the study methodology of FAMAS, including the design, sample techniques and clinic and research protocols used. To date there is little data on the factors associated with participation in the few longitudinal studies that specifically investigate the health of men in Australia. Research from other ageing cohort studies suggest that time constraints, low socio-economic status, smoking status and a lack of perceived benefit are all significant barriers to participation in men [10-12]. This is an area requiring further investigation in the Australian male population. Potential sources of selection and observational bias will be also examined through analysis of sampling and attrition. Finally, the cohort will be compared with available national data on key demographics for comparability with the target populations.

## Methods

### Study design

The Florey Adelaide Male Ageing Study (FAMAS) is a population-based longitudinal study that commenced in 2002 involving 1195 men aged 35–80 years recruited from the north-west regions of Adelaide. As a result of funding availability men were enrolled in two phases: from August 2002 until July 2003, inclusive (Phase 1, 568 participants) and June 2004 to May 2005 (Phase 2, 627 participants). Participants are asked to complete annual follow-up questionnaires tracking any changes to their contact, health status and health service utilization. In addition, other issues such as gambling activities, sleep structure, personal relationships and personal stressors are explored. Subjects are also invited to participate in additional nested case-control studies with selected collaborators. Follow-up full clinic evaluations are scheduled to occur at five-yearly intervals.

#### Ethics & participant feedback

All protocols herein were approved by the Royal Adelaide Hospital Research Ethics committee and, where appropriate, the Aboriginal Health Research Ethics Committee of South Australia.

Participants were provided with feedback upon completion of all study procedures. Following baseline clinics, a copy of all relevant results (laboratory, clinical) accompanied with an explanatory cover letter was sent to participants' and, where permission was given, to their nominated physician. In the case of a clinically significant result, participants were advised to immediately contact their treating physician for further examination.

### Participant recruitment

#### Participant criteria & sampling

Participants in the study were required to be male, aged between 35 and 80 years at the time of recruitment, living in the defined catchment area of north and west Adelaide with a connected telephone and number listed in the Electronic White Pages (EWP), be willing and able to comply with the protocol and give written, informed consent. Exclusion criteria were limited to living outside the catchment area and telephone numbers that belonged to non-residential properties (i.e. businesses, institutions and residential-care facilities) in accordance with the desire to accurately reflect the male population of the sample. Highly trained recruitment staff were also instructed to exclude respondents if they were: a) of insufficient mental or physical ability to understand the requirements of participation or adequately participate; b) to ill or otherwise incapacitated to attend clinics; c) currently residing in an institution (e.g. aged care facility); or d) had severely limited English (see also: *Recruitment & CATI survey*).

The sample was stratified into the two health regions directly under investigation: Western Adelaide and Northern Adelaide. The northern and western areas of Adelaide comprise approximately half of the city's population and over a third of the State's population. These regions broadly reflect the demographic profile of the State's population.

Residential households were selected at random, with the male person aged between 35 and 80 years to last have his birthday invited for interview and study participation. This method of randomly selecting within the household avoids a selection bias towards the unemployed, retired or homemakers [10,12].

#### Recruitment & CATI survey

In accordance with established mailing protocols [13], a letter introducing the study, along with an information brochure, was sent to selected households approximately 2 weeks prior to attempting to contact the residence. The letter and brochure informed potential participants of the purpose of the study and indicated that they could expect to be contacted by telephone. Contact details were also supplied for willing participants who for logistical reasons, could not be contacted during regular recruitment period hours. A number of initiatives were undertaken to increase general awareness of the study in the target community. These included local media events (television, print and radio) and a study launch held at a national sporting complex and opened by the State's health minister, with various political, sporting and business identities and members of the general public attending.

The telephone recruitment was conducted by an external agency with qualified staff utilizing a Computer-Assisted Telephone Interviewing (CATI) system. This method utilises the Electronic White Pages (EWP) as the sampling frame, using six digits of the standard eight digit telephone number in addition to prefixes and exchanges provided by the directory's administrator (Telstra) within geographically defined areas. This technique yields a final sampling frame that is more than adequate to cover all households within the catchment areas and has been demonstrated to be as effective as other survey methods [14]. The CATI transcript included a series of questions relating to the interviewees demographics (age group, residential location, predominant occupation, number of adults/children in household), history of health conditions/events (physician nominated diabetes, asthma, bronchitis, emphysema, heart attack, stroke, angina or none) and nominated risk factors (smoking, weight/height self-estimates, hypercholesterolemia, hypertension). The transcript also allowed the coding of all reasons for non-participation (i.e. poor to no English skills, too busy, lack of perceived benefit, too old, don't want to, too sick, none given or other).

Following removal of all non-residential telephone numbers from the drawn sample, calls were made on alternate evenings and weekends to maximize chance of contact. Calls were also made on other occasions if specifically requested. In general, no more than ten attempts were made to the same phone number. Upon contacting the household, the interviewer firstly identified themselves and the purpose of the study. The interviews were conducted in English however every attempt was made to be as inclusive as possible for all interviewees. When required for poor-English speaking interviewees, a friend or family member of the interviewee was arranged to join the telephone interview as an interpreter (and attend the subsequent clinic session). To further facilitate recruitment, the interview was restricted to approximately 15 minutes duration. Participants were subsequently given reminder calls on the eve of their clinic visit. The period between screening call and clinic was generally within a fortnight and no more than two months

### Measurements

#### Clinic visits

Baseline visits took place at the Queen Elizabeth (TQEH) and Lyell McEwin Health Service (LMHS) depending on a participants residence. In general, clinics were held from Monday to Saturday (between 0700 and 1130) on alternating weeks at the respective locations. Participants arrived following an overnight fast of approximately 12 hours for a blood draw. If a subject's medication regimen prevented a fasting visit, this was duly recorded in the clinic notes.

Prior to participation, subjects were sent a clinic pack containing all study documentation (information & consent forms, personal, secondary & physician contact detail forms) as well as the self-administered FAMAS Questionnaire A. This was compiled as a general health and wellbeing questionnaire with well-validated measures extensively used in population research. Questionnaire A included standard demographic questions based on those in the Australian Census 2001 [15] regarding ethnicity, income, education and work status, and health information regarding medical conditions, prior surgery, medication use and cigarette smoking from other statutory sources [16]. Also included in this questionnaire was the 36-item short-form health survey (SF-36, [17]), the Beck Depression Inventory (BDI, [18]), physical activity measure (1999 National Physical Activity Survey, [19]), the International Prostate Symptom Scale (IPSS, [20]) and items assessing symptoms of obstructive sleep apnoea (OSA, [21]). Clinic packs also included the Australian Cancer Council of Victoria's (ACCV) self-administered, optically scanned Food Frequency Questionnaire (FFQ), used to assess the composition of participants' diets [22].

During clinic visits, a separate questionnaire assessing sexual desire and erectile function (Questionnaire B) was completed in private. This included the Sexual Desire Inventory 2 (SDI-2, [23]), the International Index of Erectile Function (IIEF, [24]) and a Global Impotence Rating (GIR, [25]). Participants were also required to complete a brief survey recording their levels of engagement with a variety of health care providers and satisfaction with available services (Health Service Utilization Questionnaire; see Additional File 1).

Anthropometry (height, weight, waist and hip measurements as per Norton & Olds [26]), blood pressure measurements, a brief neuropsychological assessment (Fuld Object Memory Evaluation [27], Trail Making Test [28] and finger tapping & handgrip strength [29]), and uroflowometry tests were also completed during the approximate 45-minute clinic visit (see below). In addition, clinic staff had the capacity to record any other observations on participants deemed to be of clinical relevance.

#### Blood sample

A fasting blood sample (approximately 25 ml) was taken upon arrival at clinic by venipuncture in the antecubital fussa and immediately refrigerated and transported to a NATA certified laboratory for analysis. Measured and calculated parameters are summarized in Table 1. Surplus serum was stored at -70°C for future analysis. An additional 5 ml of whole blood was collected for DNA analysis.

**Table 1:** Laboratory measurements

Laboratory Measurement	Analytic Method
**Androgens/Steroids**	
Total Testosterone (TT)	Immulite 2000
Bioavailable Testosterone (BT)	as per O'Connor et al., 1973
Sex Hormone Binding Globulin (SHBG)	Immulite 2000
Insulin-like Growth Factor (IGF-1; IGF-BP3)	

**Lipids**	
Total Cholesterol	Olympus AU5400
High Density Lipoprotein Cholesterol (HDL)	Olympus AU5400
Low Density Lipoprotein Cholesterol (LDL)	Olympus AU5400
Total Triglycerides	Olympus AU5400

**Glucose**	
Glucose	Olympus AU5400
Haemoglobin A1c (HbA1c)	Biorad Variant II
Insulin	Abott Axsym

**Prostate**	
Prostate Specific Antigen (PSA)	Abott ARCHITECT^©^

**Reproductive**	
Follicle Stimulating Hormone (FSH)	Abott ARCHITECT^©^
Luteinizing Hormone (LH)	Abott ARCHITECT^©^
Oestradiol (E_2_)	Immulite 1

**Thyroid**	
Thyroid Stimulating Hormone (TSH)	Abott ARCHITECT^©^
Triiodothyronine (T_3_)	Abott ARCHITECT^©^
Thyroxine (T_4_)	Abott ARCHITECT^©^

**Liver**	
Liver Function Test (including: Bilirubin, GGT, ALP ALP, ALT, AST, LD)	Olympus AU5400

**Kidney**	
Urea	Olympus AU5400
Creatinine	Olympus AU5400
Urate	Olympus AU5400
Phosphate	Olympus AU5400

**Blood**	
Complete Blood Exam (including: Haemoglobin, Haemodynamics, Platelets, White Cell Count)	Sysmex 1000i
Electrolytes (Sodium, Potassium, Chloride, Bicarbonate)	Olympus AU5400
Protein (including: Albumin, Globulin)	Olympus AU5400
Calcium (Total & calculated Ionized)	Olympus AU5400

Immulite 1 & Immulite 2000:Diagnostic Products Corporation, Los Angeles, Ca.;

Abbott Axsym & Abbott Architect: Abbott Diagnostics, Abbott Park Illinois; Olympus AU5400: Olympus Optical, Shizuoka-ken, Japan; Biorad Variant II: Biorad, Hercules, Ca.; Sysmex 1000i: Sysmex, Kobe, Japan.

#### Uroflowometry

A portable uroflowometry analyser (UROCAP-II, Laborie Medical, Technologies, Ontario, Canada) was used to measure multiple characteristics of a participant's urine flow (peak & mean flow; voiding & flow time; time to peak flow; voided volume). Following completion of testing, a small sample (approximately 5 ml) was collected and stored for later analysis.

#### DEXA scans

Participants had whole body and lumbar spine bone mineral density (BMD) and whole body and regional body fat and lean mass measured by dual energy x-ray absorptiometry (DEXA) at their earliest convenience using the LUNAR DPX+ pencil beam densitometer (GE Lunar Corporation). Participants were informed of radiation exposure levels (~0.18 μSv per scan) and given the opportunity to discuss the procedure with an experienced administrator. Ultimately, 89.5% of participants agreed to a DEXA scan, with work commitments being the major reason cited for non-attendance.

#### HIC data

Following specific consent from participants, data were obtained from Medicare Australia on participants' usage of the Medicare & Pharmaceutical Benefits Schemes and linked with self reports of health conditions, health service and medication usage. This data is anticipated to be collected at every clinic wave.

#### Follow-up questionnaires (FUQ)

Study participants are also required to complete annual questionnaires documenting any changes to their contact and physician details as well including repeated measures on health and physical status. In addition to repeated measures, stable variables (e.g. birth weight) are regularly included in questionnaires to better characterize the cohort. FUQ's are expected to take no more than 15 minutes to complete. FUQ's are mailed to participants at the end of the week corresponding to their initial clinic visit. After a two-week period all participants with outstanding questionnaires are contacted by phone and asked to complete the questionnaire over the phone. If contact cannot be made, or if the participant does not have time, attempts are made to make contact the participant the following week for up to six weeks (assuming the participant remains willing). If after this period contact cannot be made, participant details are sought through all available secondary contacts (including nominated physicians) until notification is received. All contact with participants is logged into the study database.

#### Sub studies

In addition to the primary research aims of FAMAS, a number of sub studies have commenced in collaboration with researchers from various fields. The sub-studies are run in parallel with the main study. For a particular sub-study, the enrolment criteria are defined, potential participants identified from the database, and a letter is sent with an invitation to enrol, with a follow-up telephone call after one week. Projects were required to be broadly related to the aims of FAMAS, to be either of informative or health value to participants and to avoid excessive contact. These have been detailed previously [30].

### Cohort retention strategies

Several previously successful strategies in maximizing cohort retention and maintaining regular contact were adopted. These included study branding for ease of recognition in the general and research community, explaining longitudinal nature of research, using experienced and friendly staff during clinics, placing emphasis on the potential direct and indirect health benefits to participants and the broader community, detailed contact information whilst emphasising confidentiality measures, use of reply paid envelopes for all source documentation and refunding postage fees incurred by participants [13,31]. Other measures adopted to maintain regular participant contact and ascertain current status included: i) *Birthday & Christmas Cards *(also served to improve participant compliance); ii) *Biannual Newsletter *(an additional avenue to disseminate study results and general information); iii) *Follow-up Questionnaires *(a primary data source that includes participants' personal, secondary and health provider details).

A strict series of guidelines were also adopted for participant withdrawal and deaths. In the case of death, mortality data is also monitored from statutory sources (e.g. S.A Births, Death & Marriages Registration Offices, S.A. Cancer Registry, & National Death Index).

### Representativeness

The representativeness of the cohort was assessed through comparison of selected demographic data (age, region, marital status, educational data, income & work status) with the 2001 Census figures [15] for the target (northern & western Adelaide) and Australian population.

### Data collection & analyses

All study data collected is checked for completeness and clarity upon receipt and stored on a secure SQL server, backed up nightly and accessed through a customized management information system. All source and linked data undergo random quality control checks for corruptions during data migration.

For responders vs. non-responders, and active participants vs. withdrawn, groups were compared using chi-square tests and the resulting standardized cell residuals for categorical characteristics and t-tests for continuous characteristics, where appropriate.

## Results

### Response rates

**Figure 1:**
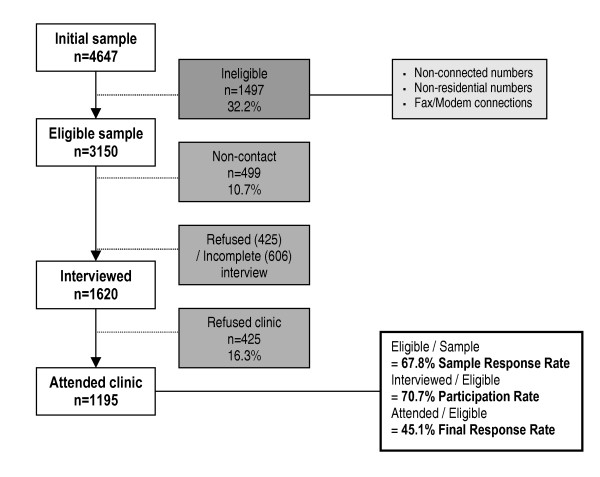
Participant disposition.

Figure 1 shows the participant disposition from initial random sampling through to participation in study clinics. After adjusting for those not contactable or ineligible in accordance with the methods of Slattery et al [32], the response rate for the study (percentage of sample eligible for recruitment) was 67.8%, the overall participation rate (percentage of eligible sample who agreed to be interviewed) was 70.7% and the final response rate of the eligible sample that ultimately attended the clinic was 45.1%. Of the 3115 men sampled, 57 had "low English" or equivalent recorded.

### Non- responders

A comparison between non-responders and the 1195 study participants is shown in Table 2. Non-responders were those men that refused participation in the study but had completed some or all of a series of supplementary demographic questions.

**Table 2:** Selected characteristics of FAMAS participants & non-responders

Characteristic	Participant	Non-Responder	*p*-value
**Region:**	**[n = 1195]**	**[n = 1920]**	**0.12**
Western Adelaide	589 (49.3)	1002 (52.2)	
Northern Adelaide	606 (50.7)	918 (47.8)	

**Age Group:**	**[n = 1195]**	**[n = 487]**	**0.40**
35–44	271 (22.7)	125 (25.7)	
45–54	326 (27.3)	136 (27.9)	
55–64	305 (25.5)	108 (22.2)	
65–80	293 (24.5)	118 (24.2)	

**Height (cm): mean (sd)**	**[n = 1195]**	**[n = 556]**	**0.81**
	175.8 (7.2)	175.7 (7.4)	
**Weight (kg): mean (sd)**	**[n = 1194]**	**[n = 529]**	**0.26**
	85.1 (14.7)	84.2 (15.7)	
**BMI (kg/cm^2^): mean (sd)**	**[n = 1194]**	**[n = 518]**	**0.35**
	27.5 (4.4)	27.3 (4.6)	
**BMI Category:**	**[n = 1194]**	**[n = 518]**	**0.33**
Underweight (< 20)	25 (2.1)	9 (1.7)	
Normal (20–24.9)	324 (27.1)	162 (31.3)	
Overweight (25–30)	556 (46.6)	234 (45.2)	
Obese (>30)	289 (24.2)	113 (21.8)	

**Current Smoker:**	**[n = 1195]**	**[n = 576]**	**0.001**
Yes	216 (18.1)	147 (25.5)*	
No	945 (79.1)	413 (71.7)	
Occasionally	34 (2.8)	16 (2.8)	
**Ever Smoked Regularly:**	**[n = 979]**	**[n = 429]**	**0.83**
	556 (56.8)	241 (56.2)	

**Ever High Cholesterol:**	**[n = 1195]**	**[n = 572]**	**0.03**
Yes	447 (37.4)	181 (31.6)	
No	714 (59.7)	381 (66.6)*	
Don't know	15 (1.3)	6 (1.0)	
Never Measured	19 (1.6)	4 (0.7)	
**Current High Cholesterol:**	**[n = 438]**	**[n = 181]**	**0.0003**
Yes	236 (53.9)	99 (54.7)	
No	70 (16.0)	50 (27.6)*	
Don't know	132 (30.1)	32 (17.7)	

**Ever High Blood Pressure:**	**[n = 1195]**	**[n = 572]**	**0.25**
Yes	375 (31.4)	186 (32.5)	
No	816 (68.3)	381 (66.6)	
Don't know	3 (0.3)	5 (0.9)	
**Current High Blood Pressure:**	**[n = 365]**	**[n = 186]**	**0.41**
Yes	265 (72.6)	142 (76.3)	
No	77 (21.1)	37 (19.9)	
Don't know	23 (6.3)	7 (3.8)	

**No. Adults (18+) in Household:**	**[n = 1179]**	**[n = 576]**	**0.03**
1	169 (14.3)	109 (18.9)*	
2	788 (66.8)	357 (62.0)	
3	144 (12.2)	81 (14.1)	
4+	78 (6.6)	29 (5.0)	
**No. Children (<18) in Household:**	**[n = 1179]**	**[n = 576]**	**0.32**
0	862 (73.1)	407 (70.7)	
1	103 (8.7)	64 (11.1)	
2	149 (12.6)	78 (13.5)	
3	55 (4.7)	20 (3.5)	
4+	10 (0.8)	7 (1.2)	

**Physical Conditions**			
**Diabetes:**	**[n = 1195]**	**[n = 575]**	**0.0003**
	106 (8.9)*	84 (14.6)	
**Asthma:**	**[n = 1195]**	**[n = 575]**	**0.06**
	134 (11.2)	48 (8.3)	
**Bronchitis:**	**[n = 1179]**	**[n = 575]**	**0.12**
	142 (12.0)	55 (9.6)	
**Emphysema:**	**[n = 1179]**	**[n = 575]**	**0.94**
	30 (2.5)	15 (2.6)	
**Heart Attack:**	**[n = 1179]**	**[n = 575]**	**0.68**
	72 (6.1)	38 (6.6)	
**Stroke:**	**[n = 1179]**	**[n = 575]**	**0.04**
	21 (1.8)*	19 (3.3)	
**Angina:**	**[n = 1195]**	**[n = 575]**	**0.77**
	62 (5.2)	28 (4.9)	
**None of the above:**	**[n = 1179]**	**[n = 575]**	**0.95**
	781 (66.2)	380 (66.1)	

**Psychological Conditions**			
**Anxiety:**	**[n = 1195]**	**[n = 575]**	**0.15**
	29 (5.0)	43 (3.6)	
**Depression:**	**[n = 1195]**	**[n = 575]**	**0.53**
	60 (5.0)	33 (5.7)	
**Stress:**	**[n = 1179]**	**[n = 575]**	**0.73**
	55 (4.7)	29 (5.0)	
**Other:**	**[n = 1179]**	**[n = 575]**	**0.82**
	11 (0.9)	6 (1.0)	
**None of the above:**	**[n = 1179]**	**[n = 575]**	**0.76**
	1065 (90.3)	522 (90.8)	

Participants were classified as those men that completed all CATI preliminary questions and attended baseline clinics; Non-responders were those men that refused participation in the study but had completed some or all of the preliminary questions. Data presented as *n *(%) unless stated. * Denotes the category was statistically significant compared with non-responders based on standardised chi-squared cell residuals with magnitude < or >3.

There was no age difference observed between participants and non-responders. Similarly, there was no difference between groups for area of residence, estimated body mass index (BMI: calculated from self-reports of weight & height), or number of children in a household. Additionally, no obvious difference existed in the type of work men had done for most of their lives (data not shown).

In terms of health status, non-responders were no different in prevalence of physician-diagnosed incidences of hypertension, asthma, bronchitis, emphysema, heart attacks, episodes of angina or mental health conditions (anxiety, depression, stress or other). However, there was an increased prevalence of diabetes and stroke in those that chose not to participate. There was no recorded difference between groups for participants reporting an absence of existing health conditions. Non-responders were, however, more likely to live alone and be current smokers (although there was no difference between groups on whether they had previously smoked regularly). Non-responders were also less likely to report elevated cholesterol levels (both previously and at time of interview).

### 3.3 Loss to follow-up

In order to assess the non-randomness of any loss to follow up (LTFU) for the study to date, a comparison of selected demographics (age, employment & marital status, education & income) between active FAMAS participants and study losses was performed (Table 3). Subject losses (deaths, withdrawals) were more likely to occur in the elderly subset. No other differences in the selected demographics were observed. Of all study withdrawals, 39.5% were as a result of death. The most commonly cited causes for retraction of consent were "increased time demands" (27.3%) and "lack of interest" (13.1%). Allowing for deaths, withdrawals and non-contactable participants, the current annual loss rate for the study is only 1.1%.

**Table 3:** Demographic characteristics of study withdrawals

Demographic	Active Participants	Withdrawn	*p *value
**Age Group**:	**[n = 1155]**	**[n = 40]**	**0.04**
35–44	267 (23.1)	4 (10.0)	
45–54	318 (27.5)	8 (20.0)	
55–64	293 (25.4)	12 (30.0)	
65–80	277 (23.9)	16 (40.0)*	

**Marital Status**:	**[n = 1153]**	**[n = 40]**	
Married/Living With a Partner	940 (81.4)	34 (85.0)	
Separated/Divorced	124 (10.7)	2 (5.0)	
Widowed	32 (2.8)	1 (2.5)	
Never Married	57 (5.0)	3 (7.5)	

**Employment Status**:	**[n = 1155]**	**[n = 40]**	
Employed-full time	587 (50.8)	10 (25.0)	
Employed part-time	109 (9.4)	2 (5.0)	
Employed-self employed/not stated	61 (5.1)	6 (15.0)	
Unemployed	31 (2.7)	1 (2.5)	
Not in work force	367 (31.4)	21 (52.5)	

**Education**:			
**Any Qualification Post School:**	**[n = 1149]**	**[n = 39]**	
Yes	827 (71.6)	23 (57.5)	
No	322 (27.9)	16 (40.0)	
**Highest Qualification**:	**[n = 822]**	**[n = 23]**	
Bachelor degree or higher	141 (17.0)	1 (4.2)	
Trade/Apprenticeship	378 (45.5)	14 (58.3)	
Certificate/Diploma	256 (30.8)	7 (29.2)	
Other	47 (5.7)	1 (4.2)	

**Gross Annual Household Income**	**[n = 1134]**	**[n = 40]**	
Up to $12,000	77 (6.7)	3 (7.5)	
$12,001 – $20,000	151 (13.1)	13 (32.5)	
$20,001 – $30,000	157 (13.6)	8 (20.0)	
$30,001 – $40,000	131 (11.3)	2 (5.0)	
$40,001 – $50,000	132 (11.4)	4 (10.0)	
$50,001 – $60,000	142 (12.3)	3 (7.5)	
$60,001 – $80,000	153 (13.3)	3 (7.5)	
More than $80,000	191 (16.5)	4 (10.0)	

Study withdrawals were those participants lost to follow-up (withdrawn consent, death, lost contact) since first enrolment (Aug 2002). Data presented as *n *(%) unless stated. * Denotes the category was statistically significant compared with active participants based on standardised chi-squared cell residuals with magnitude < or > 3.

### 3.4 Demographics of study participants

Demographic characteristics of FAMAS participants, and where appropriate, corresponding Census data are shown in Table 4.

**Table 4:** Demographic profile of FAMAS participants.

	FAMAS		Census – NW Adel		Census – Aus	
	(n)	(%)	(n)	(%)	(n)	(%)
**Age**						
35–44	271	22.7*	40,931	32.4	1,409,361	32.0
45–54	326	27.3	34,993	27.7	1,276,302	29.0
55–64	305	25.5*	24,271	9.2	884,786	20.1
65–80	293	24.5	32,409	20.6	835,577	19.0

**Region of Birth**						
Oceania	806	67.4	188,475	70.7	6,939,747	74.9
Australia	795	66.5	186,324	69.9	6,712,876	72.4
Other	11	0.9	2,151	0.8	226,871	2.4
Europe	345	28.9	49,510	18.6	1,077,073	11.6
UK &Ireland	218	18.2	26,230	9.8	545,146	5.9
Other	127	10.6	23,280	8.7	531,927	5.7
Asia	29	2.4	10,392	3.9	57,172	4.9
Africa	7	0.6	2,410	0.9	183,236	2.0
Americas	6	0.5	1,497	0.6	78,612	0.8
Other	0	0.0	216	0.1	8,653	0.1

**Marital Status**:						
Married or Living With a Partner	974	81.5	86,567	68.6	3,118,768	64.1
Separated/Divorced	126	10.5	19,891	15.8	623,689	16.6
Widowed	33	2.8	3,607	2.9	116,676	3.2
Never Married	60	5.0*	16,142	12.8	546,893	16.1

**Employment Status**:						
Employed-full time	597	50.0	71,567	48.5	2,210,528	47.9
Employed-part-time	111	9.3	13,681	9.3	477,007	10.3
Employed-self employed/not stated	42	3.5	1,795	1.2	77,849	1.7
Unemployed	32	2.7	5,992	4.1	173,473	3.8
Not in work force	405	33.9	50,555	34.2	1,474,897	32.0

**Education**:						
**Any qualification post-school?**						
Yes	850	71.1	46,025	35.8	2,977,559	41.0
No	338	28.3	71,721	55.7	3,532,760	48.6
**Non-school qualification**:						
Bachelor or higher	142	24.2	7,643	13.4	910,318	24.4
Trade	392	66.8	38,382	67.4	2,067,241	55.4
Other	48	8.2	10,907	19.2	752,225	20.2

Census data was obtained from the Australian Bureau of Statistics' Basic Community Profiles for Australian, Northern Adelaide and Western Adelaide Statistical Divisions. Census data on employment status is available for all household males in the respective divisions aged 35 years or over. Census data on education includes all household males aged 15 years or over. * Denotes the category was statistically significant compared with Census 2001 data using standardised chi-squared cell residuals with magnitude < or > 3.

#### Age

Table 4 shows the age distribution of the cohort. The mean age of the study participants was 55.0 ± 11.6 (min. 35 – max 80 at clinic). Residual analysis from a goodness-of-fit test demonstrated that young males (<45) were under represented and 55–64 years old were over represented in comparison to Census data (data not shown).

#### Marital status

Eighty-two percent (N = 974) of men were married or living with a partner. In comparison to Census data, there was an under representation of men who had never married (Table 4).

#### Region of birth

Sixty-seven percent (N = 795) of participants were born in Australia, slightly higher proportions than observed in both Census figures (Table 4). The most frequent countries of birth outside of Australia were the United Kingdom (including North Ireland, Scotland and Wales) & Ireland with 218 participants (18.2%). Such participants appeared to be overrepresented in the study sample as compared to the broader population. Of the men born outside Australia, the average amount of time spent in Australia was 36.5 ± 11.9 years.

#### Employment status

Fifty percent (N = 597) of participants were in full-time employment; 9% (N = 111) part-time and 4% (N = 42) self-employed, whilst 3% (N = 32) were unemployed at time of survey. Thirty-four percent (N = 405) had no active involvement with the work force, the majority of whom were retired (N = 351). In general, there was good agreement between study and Census figures of employment (Table 4).

#### Education level

Seventy-one percent (N = 848) of men had obtained some form of qualification since leaving school (Table 4). Sixty-seven percent (N = 392) had obtained a trade qualification or equivalent, 24% (N = 142) had a bachelor degree or higher whilst 8% (N = 48) reported having some other qualification (Table 4). The average age that participants left school was 16.0 ± 2.0 years old. When compared to Census figures, which include all males over the age of 15 years, the FAMAS cohort appears to display a higher proportion of study participants with some form of post-school qualification; specifically, a higher proportion of trade & tertiary qualifications were observed in the cohort when compared to North West Adelaide and Australian males, respectively (Table 4).

#### Gross annual household income

**Figure 2:**
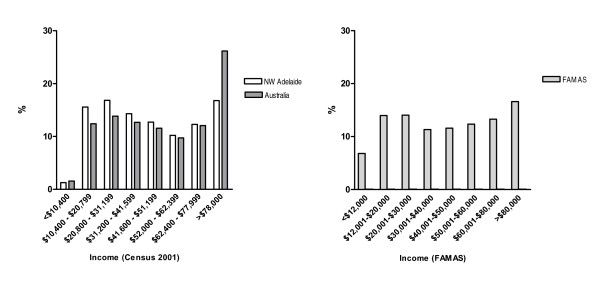
**Comparison of annual incomes between Census 2001 data and FAMAS participants**. Available Census 2001 income figures (Average Weekly Family Income) were altered to approximate collected annual gross household data. End income brackets were multiplied by 52; reported family income data was assumed to reflect gross household income.

For an approximate comparison, Census income data (Average Weekly Earnings) were extrapolated into annual figures. Of the 98% of study participants who disclosed their gross annual household income, 7% (N = 80) of men had gross household incomes in the lowest bracket (up to $12,000 p.a.) (Figure 2) which appeared to be a higher proportion than that observed in the target population. The remaining income brackets in the cohort were consistent with the distribution observed in the broader populations (Figure 2), with the noted exception of an absence of the high-income spike observed in the Australian data.

## Discussion

The population sampled for the Florey Adelaide Male Ageing Study (FAMAS) has yielded participants broadly representative of the sampling region and the broader Australian population of Australian men aged 35–80 in key demographics. Efforts will now focus on maintaining participation in a unique and expanding investigative base.

All longitudinal studies must include an assessment of their baseline response rates [33]. Studies with low levels of initial recruitment can potentially introduce significant levels of selection bias into the sample. Response rates can be difficult to interpret as the methods used to calculate them are often not specified. Some studies report response rates as a percentage of eligible people in the sample, whilst others report it as a percentage of the entire sample. The former often leads to much higher rates. Our chosen method concurs with that most often used in research of this type [33]. By way of comparison, our observed response rates (sample: 67.8%; clinic: 45.1%) appear much higher than those of another study (clinic response rate: 29%) using very similar sampling procedures [34]. Certainly given the study type and investigations (including detailed sexual health measures), the observed response rates seem sufficient.

Several other studies have employed additional methods to bolster their recruitment rates (see [32] for review). These have included the use of "volunteer enrichment" (e.g. Framingham Heart Study, [35]), whereby investigators allowed unsolicited participants to enter the study. Whilst many approaches from suitable candidates were received during promotional activities for the study, such requests were declined in favour of maintaining a randomly-selected sample. We have, however, managed to achieve reasonable levels of initial recruitment from our target population by minimizing exclusion criteria and incorporating measures in our study design that would appeal to our target population. Indeed, most studies [36,37] report that participation seems largely related to the perceived benefits of participation. Thus, measures such as a comprehensive health review and relatively low contact time appear to have helped increase involvement in a population traditionally hesitant to participate [38,39]. Despite this, the most commonly cited reasons for study refusal were householders being either 'too busy' or 'not interested', universal responses in any study of this type [40]. Interestingly, people citing being 'too old', 'too sick' or for having 'no health problems' were only minor contributors to study refusal, in contrast to previous findings [41,42]. Whilst the reasons given for refusing participation in health research have been well documented elsewhere, there is a scarcity of such data from Australian male cohort studies. Such findings will have implications for future studies and preventative programmes involving ageing men.

Comparisons of non-responders and study participants in any study is useful in highlighting key points of difference between the two groups and allowing for an estimation of any sources of selection bias. The under representation of single men in the cohort provides further agreement with a volume of research [43] that suggests that people (especially the elderly) with live-in support systems generally demonstrate higher levels of attendance in health studies (and, more generally, better health outcomes) than people who live alone. There is also strong evidence to suggest that non-responders tend to display poorer health outcomes (e.g. an increased incidence of strokes [44] and diabetes [45]). However, in the present study non-participators were less likely to have had previous and current episodes of hypercholesterolemia. During pre-recruitment promotion, strong emphasis was placed on the free cholesterol testing and, combined with a recent national emphasis on elevated cholesterol levels, this may have dissuaded men who had previously or were currently receiving treatment. In all, our analyses do suggest that our study participants are broadly representative of the sampled population and that those that were unwilling to participate did not create a substantial bias. However, the observed discrepancies will have to be taken into account when applying study results to the overall population.

In any longitudinal design, particular effort is needed in characterizing loss to follow up (LTFU) in order to help identify potential sources of future cohort loss [46]. Previous research has identified a host of demographic factors that are associated with LTFU (see [31] for review). In most population studies, those greater than 60 years are less likely to withdraw [47], although this is likely offset by an elevated death rate. Indeed, in the present study, almost half of all study losses were as a result of participant death, the large majority of which came from the initial 65–80 age bracket. Aside from age, no other demographic factor appears to have influenced LTFU. The current LTFU rates are extremely low in comparison to other studies of this type [4]. Whilst it is acknowledged that our second wave of clinic visits is yet to begin, we have none the less been able to maintain a good rate of contact with most of the cohort to date through a host of activities. Although efforts to maximize participation appear to have been effective, other successful measures adopted by similar studies (especially in the critical elder and younger brackets [47-49]) will also be utilized to maintain the stability of the cohort.

Whilst the ability to generalize results from a given study to the broader population is one of the key elements of longitudinal research it is quite often not the primary focus of the study. Certainly many of the more noted cohort studies (e.g. British Doctors Survey [50], CARDIA [51], Framingham [52]) were non-representative by design. In the present study however a broadly representative cohort was sought. As argued by Szklo [33], an important determinant in realizing a representative cohort is the selection of a complete sampling frame. In this context, our use of the Electronic White Pages may be seen as a limitation given that this restricts potential respondents to those with a connected line. However, research [14,53] using a comparable sampling technique, has suggested that this is not the case.

In terms of the age profile of our cohort, the lower proportion of young males in the cohort is notable. This is a common phenomenon in studies with similar sampling designs [31,53] and a number of initiatives to maximize the ongoing involvement of this group of men have been adopted in consequence (e.g. family days, targeting specific media outlets). The overrepresentation of the elderly has also been observed previously, and whilst the reasons for this are situation-dependant, it is generally considered that such participants can offer more time to community-based research [54]. Whilst the profile of FAMAS participants did significantly differ from the broader community in selected age groups, the careful application of appropriate weights to future results should offset any age-influenced bias. Additionally, the present age profile will help to ensure adequate inter-age group comparisons can be performed, an important consideration in any ageing longitudinal study. Indeed, we would argue that our cohort is well placed long-term to cope with the attrition normally associated with studies of this type.

The low numbers in some regions of birth means that a statistical comparison was not warranted. Most notably, we do appear slightly overrepresented with participants from the UK/Ireland. Whilst no clear reasons exist for this discrepancy, it is interesting to note that some of the highest response rates for population studies are recorded in British surveys [55]. The apparent lower proportions in other regions (whilst not statistical) are slightly more difficult to explain, and may reflect a general trend towards non-participation in this country [56]. Similar patterns have been observed in a nationwide telephone-based survey of the reproductive health of approximately 6000 men age 40–80 (Men in Australia: Telephone Survey; MATeS) [57,58].

The income distribution for the cohort also appears broadly reflective of the wider populations. The disparity between the proportion of high household incomes in the north west Adelaide and Australian populations is indicative of some of the socio-economic disadvantages of the region [59].

Many population studies report that a respondents level of education strongly predicts their likelihood of involvement [60,61]. This notion received support in the present study with a higher proportion of participants with some post-school qualification as compared to the general population. These figures bode well for the stability of the cohort, with previous research demonstrating that those with a higher educational standard are less likely to withdraw [62,63]. Taken together, these figures suggest that the men in the present study are generally reflective of the community from which they were randomly selected and the Australian population as a whole.

In terms of the overall study frame, the occurrence of clinics at every five years should be adequate for the conditions under investigations. Whilst other cohort studies have benefited from more frequent clinical investigations [62,63] these studies were investigating inherently dynamic disease conditions or outcomes. In addition to the logistical and financial expense associated with clinics, participant contact beyond that which is necessary is likely to lower recruitment and increase LTFU. Also, in the present study there is a sustained effort to keep regular contact with participants in between clinic waves through annual follow-up questionnaires, sub-study investigations and cohort maintenance initiatives. In comparison to other longitudinal studies of ageing the incorporation of a "younger" subset is quite unique [4] and, provided these numbers can be maintained, will provide an invaluable opportunity to study the progression of various conditions and changing behaviours in men.

An *a priori *target of around 1200 participants was selected before recruitment ensuring sufficient power for the main outcome variables. Even relatively minor correlations between outcomes could be reliably detected. Whilst this cohort size may be smaller in comparison to other longitudinal studies of ageing we would argue that the focus on a single gender and the high stability of the cohort observed to date (in part due to the conscious efforts to minimize losses to follow-up) will ensure a relatively large number of active, participating subjects for the duration of the study.

The north west region of Adelaide appears an ideal community in which to investigate the ageing process, with a population that is largely reflective of the state with the highest proportion of elderly in Australia. The urban-specific focus of the study may limit the broader applicability of study results, however in the present circumstance incorporating regional participants was not feasible. Also, at present there are no home visits planned for participants with limited mobility given the logistics and expenses involved. There is still considerable debate on whether such measures can reduce the high attrition associated with elderly participants in cohort studies [64].

## Conclusion

The Florey Adelaide Male Ageing Study will provide an opportunity to add to existing ageing longitudinal research, both internationally and locally, with a well-characterized, broadly representative cohort that remains largely active. The study design reflects a growing trend of integrative research on ageing with inputs from a range of disciplines (endocrinology, epidemiology, gerontology, public health, psychology, sociology, ophthalmology, bone and joint disease, nutrition, genomic health, politics). The inclusion of middle aged participants is unique relative to most ageing longitudinal studies, and will allow the detection of earlier life trajectories and morbidity factors associated with healthy ageing in later life. Of the few longitudinal studies specific to conditions affecting men in Australia, most are characterised as specialized investigations of the late elderly, limiting the scope of problems addressed and the potential as a longitudinal study. By contrast, FAMAS offers a broad-based approach to many of the conditions of the ageing male, increasing acknowledged as areas that require significant research input.

## Competing interests

The author(s) declare that they have no competing interests.

## Authors' contributions

GAW & MTH conceived of the study. SAM participated in the design, management and coordination of the study and drafted the manuscript. GAW acts as Chief Investigator and continues to manage the study. MTH participated in the study design and coordination. SMM performed statistical analyses. All of the authors read and approved the final manuscript.

## Pre-publication history

The pre-publication history for this paper can be accessed here:



## Supplementary Material

Additional file 1 Health Service Utilization Questionnaire Click here for file

## References

[B1] Australian Institute of Health and Welfare (1999). Older Australia at a glance (AIHW Cat No AGE 12) Canberra.

[B2] Commonwealth Department of Health and Ageing (2001). Population, Ageing and the Economy (Research by Access Economics) Canberra.

[B3] Prime Ministers' Science, Engineering and Innovation Council (2003). Proming Healthy Ageing in Australia Canberra.

[B4] Logie H, Hogan R, Peut A (2004). Longitudinal studies of ageing: Implications for future studies.

[B5] Fletcher R (1995). Testosterone Poisoning or Terminal Neglect? The Men's Health Issue. Parliamentary Research Service (Research Paper No 22).

[B6] Jacomb PA, Jorm AF, Korten AE, Rodgers B, Henderson S, Christensen H (1997). GP attendance by elderly Australians: evidence for unmet need in elderly men. Med J Aust.

[B7] Connell R, Schofield T, Walker L, Wood J, Butland D, Fisher J, Bowyer J (1999). Men's health: A research agenda and background report. Department of Health and Aged Care.

[B8] Australian Medical Association (AMA) Position Statement on Men's health. http://www.ama.com.au/web.nsf/doc/WEEN-6B56JJ.

[B9] Mathers C Health differentials between Australian males and females: A statistical profile. National Men's Health Conference: 10–11 August 1995 Melbourne.

[B10] Slattery ML, Edwards SL, Caan BJ, Kerber RA, Potter JD (1995). Response rates among control subjects in case-control studies. Ann Epidemiol.

[B11] Eastwood BJ, Gregor RD, MacLean DR, Wolf HK (1996). Effects of recruitment strategy on response rates and risk factor profile in two cardiovascular surveys. Int J Epidemiol.

[B12] Grant JF, Chittleborough CR, Taylor AW, Dal Grande E, Wilson DH, Phillips PJ, Adams RJ, Cheek J, Price K, Gill T, North West Adelaide Health Study Team (2006). The North West Adelaide Health Study: detailed methods and baseline segmentation of a cohort for selected chronic diseases. Epidemiol Perspect Innov.

[B13] Dillman DA (1978). Mail and telephone surveys: the total design method.

[B14] Wilson D, Starr G, Taylor A, Dal Grande E (1999). Random digit dialling and Electronic White Pages samples compared: demographic profiles and health estimates. Aust N Z J Public Health.

[B15] Australian Bureau of Statistics (2001). 2001 Census Dictionary (Publication 29010) Canberra.

[B16] Wilson D, Wakefield MA, Taylor A (1992). The South Australian Health Omnibus Survey. Health Promot J Austr.

[B17] Ware JE, Sherbourne CD (1992). The MOS 36-item short-form health survey (SF-36). I. Conceptual framework and item selection. Med Care.

[B18] Beck AT, Beck RW (1972). Screening depressed patients in family practice. A rapid technic. Postgrad Med.

[B19] Armstrong T, Bauman A, Davies J (2000). Physical activity patterns of Australian adults. Results of the 1999 Physical Activity Survey.

[B20] Barry MJ, Fowler FJ, O'Leary MP, Bruskewitz RC, Holtgrewe HL, Mebust WK, Cockett AT (1992). The American Urological Association symptom index for benign prostatic hyperplasia. The Measurement Committee of the American Urological Association. J Urol.

[B21] Maislin G, Pack AI, Kribbs NB, Smith PL, Schwartz AR, Kline LR, Schwab RJ, Dinges DF (1995). A survey screen for prediction of apnea. Sleep.

[B22] Hodge A, Patterson A, Brown W, Ireland P, Giles G The Anti Cancer Council of Victoria FFQ: Relative validity of nutrient intakes compared with weighted food records in young to middle aged women in a study of iron supplementation. Aust NZ J Pub Health.

[B23] Spector IP, Carey MP, Steinberg L (1996). The sexual desire inventory: development, factor structure, and evidence of reliability. J Sex Marital Ther.

[B24] Rosen RC, Riley A, Wagner G, Osterloh IH, Kirkpatrick J, Mishra A (1997). The international index of erectile function (IIEF): a multidimensional scale for assessment of erectile dysfunction. Urology.

[B25] Feldman HA, Goldstein I, Hatzichristou DG, Krane RJ, McKinlay JB (1994). Construction of a surrogate variable for impotence in the Massachusetts Male Aging Study. J Clin Epidemiol.

[B26] Norton K, Olds T (2001). Morphological evolution of athletes over the 20th centur: causes and consequences. Sports Med.

[B27] Fuld PA, Masur DM, Blau AD, Crystal H, Aronson MK (1990). Object-memory evaluation for prospective detection of dementia in normal functioning elderly: predictive and normative data. J Clin Exp Neuropsychol.

[B28] Spreen O, Strauss E (1998). A compendium of neuropsychological tests.

[B29] Lezak M (1995). Neuropsychological assessment.

[B30] Martin S, Haren M, Taylor A, Middleton S, Wittert G Cohort Profile : The Florey Adelaide Male Ageing Study (FAMAS). Int J Epidemiol.

[B31] Hunt JR, White E (1998). Retaining and tracking cohort study members. Epidemiol Rev.

[B32] Slattery ML, Edwards SL, Caan BJ, Kerber RA, Potter JD (1995). Response rates among control subjects in case-control studies. Ann Epidemiol.

[B33] Szklo M (1998). Population-based cohort studies. Epidemiol Rev.

[B34] Dunstan DW, Zimmet PZ, Welborn TA, Cameron AJ, Shaw J, de Courten M, Jolley D, McCarty DJ (2002). The Australian Diabetes, Obesity and Lifestyle Study (AusDiab) – methods and response rates. Diabetes Res Clin Pract.

[B35] Dawber TR, Meadors GF, Moore FE (1951). Epidemiological approaches to heart disease: the Framingham Study. Am J Public Health.

[B36] Eastwood BJ, Gregor RD, MacLean DR, Wolf HK (1996). Effects of recruitment strategy on response rates and risk factor profile in two cardiovascular surveys. Int J Epidemiol.

[B37] Marmor JK, Oliveria SA, Donahue RP, Garrahie EJ, White MJ, Moore LL, Ellison RC (1991). Factors encouraging cohort maintenance in a longitudinal study. J Clin Epidemiol.

[B38] Janzon L, Hanson BS, Isacsson SO, Lindell SE, Steen B (1986). Factors influencing participation in health surveys. Results from prospective population study 'Men born in 1914' in Malmo, Sweden. J Epidemiol Community Health.

[B39] Tibblin G, Aurell E, Hjortzberg-Nordlund H, Paulin S, Risholm L, Sanne H, Wilhelmsen L, Werkoe L (1965). A General Health-Examination of a Random Sample of 50-Year-Old Men in Goeteborg. Acta Med Scand.

[B40] Thorogood M, Coulter A, Jones L, Yudkin P, Muir J, Mant D (1993). Factors affecting response to an invitation to attend for a health check. J Epidemiol Community Health.

[B41] Wall M, Teeland L (2004). Non-participants in a preventive health examination for cardiovascular disease: characteristics, reasons for non-participation, and willingness to participate in the future. Scand J Prim Health Care.

[B42] Jacomb PA, Jorm AF, Korten AE, Christensen H, Henderson AS (2002). Predictors of refusal to participate: a longitudinal health survey of the elderly in Australia. BMC Public Health.

[B43] Cape RD, Gibson SJ (1994). The influence of clinical problems, age and social support on outcomes for elderly persons referred to regional aged care assessment teams. Aust N Z J Med.

[B44] Li C, Engstrom G, Hedblad B, Berglund G, Janzon L (2005). Risk factors for stroke in subjects with normal blood pressure: a prospective cohort study. Stroke.

[B45] Engstrom G, Hedblad B, Nilsson P, Wollmer P, Berglund G, Janzon L (2003). Lung function, insulin resistance and incidence of cardiovascular disease: a longitudinal cohort study. J Intern Med.

[B46] Zunzunegui MV, Beland F, Gutierrez-Cuadra P (2001). Loss to follow-up in a longitudinal study on aging in Spain. J Clin Epidemiol.

[B47] Mihelic AH, Crimmins EM (1997). Loss to folow-up in a sample of Americans 70 years of age and older: The LSOA 1984–1990. J Gerontol B Psychol Sci Soc Sci.

[B48] Psaty BM, Cheadle A, Koepsell TD, Diehr P, Wickizer T, Curry S, VonKorff M, Perrin EB, Pearson DC, Wagner EH (1994). Race- and ethnicity-specific characteristics of participants lost to follow-up in a telephone cohort. Am J Epidemiol.

[B49] Hebert R, Bravo G, Korner-Bitensky N, Voyer L (1996). Refusal and information bias associated with postal questionnaires and face-to-face interviews in very elderly subjects. J Clin Epidemiol.

[B50] Doll R, Hill AB (1964). Mortality in relation to smoking: ten years' observations of British doctors. Br Med J.

[B51] Friedman GD, Cutter G, Donahue R, Hughes G, Hulley S, Jacobs D, Liu K, Savage P (1988). CARDIA : Study design, recruitment and some characteristics of the examined subjects. J Clin Epidemiol.

[B52] Dawber T, Meadors G, Moore F (1951). Epidemiological Approaches To Heart Disease: The Framingham Study. Am J Public Health.

[B53] Taylor AW, Wilson DH, Wakefield M (1998). Differences in health estimates using telephone and door-to-door survey methods – a hypothetical exercise. Aust N Z J Public Health.

[B54] Osler M, Schroll M (1992). Differences between participants and non-participants in a population study on nutrition and health in the elderly. Eur J Clin Nutr.

[B55] Barrett G, Cassell JA, Peacock JL, Coleman MP (2006). National survey of British public's views on use of identifiable medical data by the National Cancer Registry. BMJ.

[B56] Taylor AW, Dal Grande E, Gill T, Chittleborough CR, Wilson DH, Adams RJ, Grant JF, Phillips P, Ruffin RE (2006). Do people with risky behaviours participate in biomedical cohort studies?. BMC Public Health.

[B57] Holden CA, Jolley DJ, McLachlan RI, Pitts M, Cumming R, Wittert G, Handelsman DJ, de Kretser DM (2006). Men in Australia Telephone Survey (MATeS) : predictors of men's help-seeking behaviour for reproductive health disorders. Med J Aust.

[B58] Holden CA, McLachlan RI, Pitts M, Cumming R, Wittert G, Agius PA, Handelsman DJ, de Kretser DM (2005). Men in Australia Telephone Survey (MATeS) : a national survey of the reproductive health and concerns of middle-aged and older Australian men. Lancet.

[B59] Glover J, Hetzel D, Glover L, Page A, Leahy K (2005). Central Northern Adelaide Health Service: A social health atlas.

[B60] Krousel-Wood MA, Re RN, Abdoh A, Chambers R, Altobello C, Ginther B, Bradford D, Kleit A (2001). The effect of education on patients' willingness to participate in a telemedicine study. J Telemed Telecare.

[B61] Chinn DJ, White M, Howel D, Harland JO, Drinkwater CK (2006). Factors associated with non-participation in a physical activity promotion trial. Public Health.

[B62] Victor RG, Haley RW, Willett DL, Peshock RM, Vaeth PC, Leonard D, Basit M, Cooper RS, Iannacchione VG, Visscher WA, Staab JM, Hobbs HH, Dallas Heart Study Investigators (2004). The Dallas Heart Study: a population-based probability sample for the multidisciplinary study of ethnic differences in cardiovascular health. Am J Cardiol.

[B63] Stone JL, Norris AH (1966). Activities and attitudes of participants in the Baltimore longitudinal study. J Gerontol.

[B64] Madigan EA, Tullai-McGuinness S, Neff DF (2002). Home health services research. Annu Rev Nurs Res.

