# Supplementary material for: The Florey Adelaide Male Ageing Study (FAMAS): Design, procedures & participants
Source: BMC Public Health. 2007 Jun 27;7:126. doi: 10.1186/1471-2458-7-126 (PMC1925076; doi:10.1186/1471-2458-7-126)
Supplement: Additional file 1 — Health Service Utilization Questionnaire [file 1471-2458-7-126-S1.pdf]

Health Service Utilisation Questions

1. Have you visited a G.P (General Practitioner) in the last twelve months? (please tick box)

a) ☐ No, go to question 2

b) ☐ Yes (see below)

If yes, please indicate how many times you visited your G.P. (tick box)

☐ 1-2 Times ☐ 3-4 Times

☐ 5-9 Times ☐ 10+ Times

i) What was the main reason for your last visit to your G.P. (please tick one):

☐ Health Crisis/Sickness ☐ Health Advice

☐ Medication ☐ Regular Check-Up

☐ Screening (e.g. prostate check) ☐ Immunisation

☐ Family (e.g. children) ☐ Other (please specify)………

ii) Overall, how would you rate your last visit to the G.P. (please tick box)

☐ Excellent ☐ Very Good ☐ Good ☐ Fair ☐ Poor

iii) Were any other health concerns addressed by your doctor, other than your main health concern? (please tick box)

a) ☐ No, go to question 2

b) ☐ Unsure, go to question 2

c) ☐ Yes. If yes, did you want this health concern to be addressed (please tick box)

a. ☐ Yes

b. ☐ No

c. ☐ Undecided

2. Other Health Service Providers

i) Please indicate <u>how many times</u> you have visited the following health service providers in the last twelve months (<u>write number of visits in the box</u>)

e.g. [3] Pharmacist (indicates 3 times in last twelve months)

☐ Physiotherapist	☐ Occupational Therapist
☐ Dietitian/Nutritionist	☐ Dentist/Orthodontist
☐ Podiatrist	☐ Pharmacist
☐ Naturopath	☐ Chiropractor
☐ Osteopath	☐ Fitness/ Personal Trainer
☐ Optometrist	☐ Audiologist

☐ Nurse

☐ Medical specialist (e.g. cardiologist), please specify…………………

☐ Other, please specify………………………………………………….

ii) Indicate the three most influential health service providers with respect to your health and wellbeing (please tick no more than 3)

☐ Physiotherapist	☐ Occupational Therapist
☐ Dietitian/Nutritionist	☐ Dentist/Orthodontist
☐ Podiatrist	☐ Pharmacist
☐ Naturopath	☐ Chiropractor
☐ Osteopath	☐ Fitness/ Personal Trainer
☐ Optometrist	☐ Audiologist

☐ Nurse ☐ General Practitioner

☐ Medical specialist (e.g. cardiologist), please specify……………………

☐ Other, please specify……………………………………………………

iii) When seeking health advice, which of the settings below, do you find most welcoming? (tick no more than three)

☐ Community Health Centre ☐ Private Practice (e.g. Dr's rooms)

☐ Hospital (in-patient, e.g. surgery) ☐ Hospital (Dr's rooms)

☐ Emergency/Casualty Department ☐ Fitness Centre/Gym

☐ Service provider's home ☐ Your workplace

☐ Chemist/Pharmacy ☐ Your own home

☐ Other, please specify ……………………………………………………

3. Private Health Insurance

i) Do you currently have private health insurance

a) ☐ No

b) ☐ Yes (see below)

ii) If yes, please indicate the level of cover (tick box)

☐ Basic ☐ Comprehensive (basic with Extras)

☐ Extras only ☐ Other, please specify …………………………..

iii) What type of cover (tick box)

☐ Individual ☐ Other, please specify …………………………..

☐ Family
